# Isolation, Identification and Antimicrobial Susceptibility Pattern of *Tatumella ptyseos* Strains Isolated From Powdered Infant Formula Milk Consumed in Neonatal Intensive Care Unit: First Report From Iran

**DOI:** 10.5812/jjm.10608

**Published:** 2014-06-01

**Authors:** Jalal Mardaneh, Mohammad Mehdi Soltan Dallal, Mehrnaz Taheripoor, Zahra Rajabi

**Affiliations:** 1 Professor Alborzi Clinical Microbiology Research Center, Nemazee Hospital, Shiraz University of Medical Sciences, Shiraz, IR Iran; 2 Division of Bacteriology, Department of Pathobiology, School of Public Health, Tehran University of Medical Sciences, Tehran, IR Iran; 3 Food Microbiology Research Center, Tehran University of Medical Sciences, Tehran, IR Iran

**Keywords:** Intensive Care Unit, Neonatal, Microbial Sensitivity Tests, Powdered Infant Formula Milk, *Tatumella ptyseos*, Iran

## Abstract

**Background:**

*Tatumella ptyseos* is a rod-shaped, Gram-negative, facultative, and anaerobic bacteria categorized in the *Enterobacteriaceae* family. It is a rare food-borne opportunistic pathogen which causes neonatal sepsis, bacteremia, and urinary tract infections. *T. ptyseos* has been also cultured from various food sources around the world.

**Objectives:**

It is difficult to determine the source of the infection in the patients (especially newborns) due to low information about the epidemiology of *T. ptyseos*. The current study aimed to investigate the isolation, identification and antimicrobial susceptibility pattern of *T. ptyseos* strains from the consumed powdered infant formula milk (PIF) in hospital neonatal intensive care unit (NICU).

**Materials and Methods:**

A total of 125 powdered infant formula milk (PIF) samples were purchased from drug stores from June 2011 to March 2012. *T. ptyseos* was isolated according to food and drug administration (FDA) method. For final confirmation, biochemical testes embedded in API-20E system were used. Drug susceptibility test was performed using the disc diffusion method, according to clinical and laboratory standard institute (CLSI) recommendations.

**Results:**

Results of the study showed that, out of 125 samples, *T. ptyseos* was isolated from four (3/2%) PIF samples. All isolated strains (100%) were resistant to ampicillin, carbenicillin, cotrimoxazole and amoxicillin.

**Conclusions:**

The present study was the first report on the isolation and identification of *T. ptyseos* from PIF in Iran. *T. ptyseos* are frequently present in various kinds of foods; therefore, further investigation on these samples is required. It is necessary to track the *T. ptyseos* in a wide variety of foods and individuals especially in immunocompromised people such as human immunodeficiency virus (HIV)-positive patients to reveal the possible routes of transmission of this pathogen to humans. In addition, molecular studies are required to determine the genetic relationship between *T. ptyseos* strains isolated from different sources.

## 1. Background

*Tatumella ptyseos* is a rod-shaped (0.6-1.2 × 0.9-3.0 mm), Gram-negative, facultative, and anaerobic bacteria categorized in the *Enterobacteriaceae* family (1, 2). This organism is catalase positive, oxidase negative, non-capsulated, non-spore-forming, and cells are motile by means of polar, subpolar or lateral flagella or non-motile at 37˚C (3, 4). Therefore, this opportunistic pathogen is rather unusual among the *Enterobacteriaceae* family. *T. ptyseos*, the only species in the *Tatumella* genus, was first identified by Hollis et al. for strains formerly called “EF-9” (1, 5, 6). The DNA G + C content of *T. ptyseos* is 53-54 mol%. Phylogenetic analysis of *T. ptyseos* type strain indicates that *Tatumella* spp. forms a distinct lineage in the family well separated from other *Enterobacteriaceae* genera. *T. ptyseos* expresses the enterobacterial common antigen (ECA). 

Diaminopropane and putrescine are the major polyamines products (7-9). *T. ptyseos* grows on MacConkey agar. It produces non haemolytic, translucent and glossy colonies on blood agar plates (24 hours incubation). These colonies are sized between 0.5 to 1.0 mm in diameter with entire edges (10, 11). This organism is distinctive in production of large inhibition zone around penicillin disk (10 units) without any motility at 37˚C, but it is weakly motile at 25˚C. It has been observed that *T. ptyseos* usually has one flagellum (polar, subpolar or lateral) per bacterial cell while the other motile members of the *Enterobacteriaceae* family have peritrichous flagella distributed on the entire cell (1, 11).

*T. ptyseos* has been isolated from human and animal clinical specimens including respiratory tract, sputum, urine, stool, blood and abdominal tumor (12-14). It has been also cultured from infants' foods, beef and pineapple (15). *T. ptyseos* is a rare food-borne opportunistic pathogen and causes neonatal sepsis, bacteremia, and urinary tract infections. Risk factors, such as diabetes, leukemia, low body weight, tuberculosis, and low immunity can also make a person more susceptible to come down with *T. ptyseos* infection (1, 11, 14, 16, 17). Newborns (especially immunosuppressed patients) and elderly individuals are more susceptible to *T. ptyseos* infections. Clinical information of *T. ptyseos* infections is very limited. The patients' possible source of the infection could not be determined for the lack of information on epidemiology of this organism.

## 2. Objectives

The outbreak of any infectious disease is easiest to detect when infection source is identified. On the other hand, according to recent reports on the isolation of *T. ptyseos* from various food sources around the world, it is very useful to get the information about the pathogens surveillance, and further studies are needed, especially regarding public health importance. The current study aimed to investigate the isolation, identification and antimicrobial susceptibility pattern of *T. ptyseos* from powdered infant formula milk (PIF) consumed in hospital neonatal intensive care unit (NICU), Tehran, IR Iran.

## 3. Materials and Methods

### 3.1. Sampling

A total of 125 powdered infant formula milk (PIF) samples were purchased from hospital drug -stores from June 2011 to March 2012. In order to isolate *T. ptyseos* from samples, the PIF cans outer surfaces were sterilized with 70% ethanol (Germany, Merck), and opened under a laminar flow cabinet. Samples were taken from each product under aseptic conditions. *T. ptyseos* was isolated according to FDA method (18, 19). The samples were prepared by mixing one, 10, and 100 g of PIF with 9, 90 and 900 mL of sterile distilled water (pre-warmed to 45˚C), respectively in three Erlenmeyer flasks. They were incubated at 37˚C for 18-24 hours; when the PIF was completely mixed and dissolved. Following the incubation, 10 mL of each sample was added to 90 mL of *Enterobacteriaceae* enrichment (EE) broth medium and placed at 37˚C for 18-24 hours.

 After incubation, a loopful of the enrichment culture was streaked onto duplicate violet red bile glucose agar (VRBGA) plates and cultured at 37˚C for 18-24 hours. A total of four suspicious colonies were picked from each VRBGA plate to obtain pure culture. To detect non-lactose fermenting isolates, presumptive colonies were streaked onto MacConkey agar, and incubated at 37˚C for 72 hours. For final confirmation; biochemical testes embedded in API-20E system (Bio-Merieux, France) were used. For long term storage, the purified isolates were saved in tryptic soy broth (TSB) with 20% glycerol (Germany, Merck) at -20˚C.

### 3.2. Drug Susceptibility Testing 

Antimicrobial susceptibility test was performed using the disc diffusion method according to CLSI recommendations (20). The Antimicrobial agents (MAST, UK) used in this study were ceftazidime (30 μg), ceftriaxone (30 μg), cefepime (30 μg), piperacillin (100 μg), mezlocillin (75 μg), ticarcillin (75 μg), imipenem (10 μg), meropenem (10 μg), aztreonam (30 μg), streptomycin (10 μg), amikacin (30 μg), gentamicin (10 μg), tetracycline (3 0μg), minocycline (30 μg), chloramphenicol (30 μg), ciprofloxacin (5μg), levofloxacin (5 μg), colistin (25 μg), tigecycline (15 μg), trimethoprim-sulfamethoxazole (25 μg), ampicillin (10 μg), nalidixic acid (30 μg), carbenicillin (100 μg), moxifloxacin (5 μg), cefotaxime (30 μg), piperacillin-tazobactam (110 μg) and tobramycin (10 μg).

## 4. Results

In the present study, out of 125 tested samples, *T. ptyseos* was isolated from four (3/2%) PIF samples. The organisms were Gram negative rods and produced non-lactose fermenting colonies on MacConkey agar and purple/pink colored colonies on VRBGA agar due to the inclusion of the neutral red indicator and crystal violet in the medium. The bacteria were motile (at 25˚C), utilized citrate at 25˚C but not at 37˚C, and *T. ptyseos* was identified based on the results of other biochemical reactions (Table 1). The antimicrobial susceptibility patterns of isolated strains are presented in Table 2.

**Table 1.** Biochemical Reactions of *T. ptyseos*

Test	Reaction/Result
**Gram stain**	Gram-negative, rod
**Triple Sugar Iron agar**	Acid (yellow) slant/
	Acid (yellow) butt.
	No gas/ No H_2_S
**Catalase**	+ (weak)
**Oxidase**	-
**Motility**	
25˚C	+
37˚C	-
**Simmons Citrate’s**	
25˚C	+
37˚C	-
**Nitrate reduction**	-
**Gas from glucose**	-
**Acid from glucose**	+
**Lactose**	-
**Arabinose**	+
**Sucrose**	+
**Mannitol**	-
**Xylose**	+
**Sorbitol**	-
**Raffinose**	-
**Malonate**	-
**Adonitol**	-
**Rhamnose**	-
**Inositol**	-
**Indole**	-
**Methyl Red (MR)**	-
**Voges Proskauer (VP)**	-
**Urease**	-
**Lysine decarboxylase**	-
**Ornithine decarboxylase**	-
**Arginine dehydrolase**	-
**Phenylalanine deaminase**	+ (weak)
**Esculin hydrolysis**	-
**Gelatine hydrolysis**	-

**Table 2.** Antimicrobial Susceptibility Profile of *T. psyteos* Strains Isolated From PIF (n = 4) ^a^

Antibiotic	Sensitive	Intermediate	Resistant
**Amikacin (AK)**	4 (100)	-	-
**Ampicillin (AP)**	-	-	4 (100)
**Aztreonam (ATM)**	3 (75)	1 (25)	-
**Cefotaxime (CTX)**	2 (50)	2 (50)	-
**Gentamicin (GM)**	4 (100)	-	-
**Meropenem (MEM)**	3 (75)	1 (25)	-
**Mezlocillin (MEZ)**	2 (50)	1 (25)	1 (25)
**Moxifloxacin (MFX)**	2 (50)	1 (25)	1 (25)
**Nalidixic acid (NA)**	3 (75)	1 (25)	-
**Streptomycin (S)**	2 (50)	1 (25)	1 (25)
**Tetracycline (T)**	4 (100)	-	-
**Ticarcillin (TC)**	1 (25)	-	3 (75)
**Chloramphenicol (C)**	3 (75)	1 (25)	-
**Ceftazidime (CAZ)**	4 (100)	-	-
**Ciprofloxacin (CIP)**	3 (75)	1 (25)	-
**Cefepime (CPM)**	4 (100)	-	-
**Imipenem (IMI)**	2 (50)	2 (50)	-
**Levofloxacin (LEV)**	4 (100)	-	-
**Minocycline (MN)**	4 (100)	-	-
**Piperacillin (PRL)**	3 (75)	-	1 (25)
**Piperacillin-tazobactam (PTZ)**	3 (75)	1 (25)	-
**Carbenicillin (PY)**	-	-	4 (100)
**Tobramycin (TN)**	4 (100)	-	-
**Cotrimoxazole (TS)**	-	-	4 (100)
**Amoxicillin (A)**	-	-	4 (100)
**Colistin (CO)**	4 (100)	-	-

^a^ Data are presented in No. (%).

## 5. Discussion

*T. ptyseos* is a relatively unknown new member of the *Enterobacteriaceae* family. The natural habitat of this organism is not well understood. This opportunistic pathogen has been rarely reported as a human infection cause; there is limited information about this organism in the medical literature. This microorganism has been implicated in tracheobronchial /pulmonary infections such as pneumonitis, asthmatic bronchitis, pharyngitis, Wegener granulomatosis, pneumonia, chronic lung disease, pulmonary edema, pulmonary tuberculosis, and gastrointestinal infection (1, 3, 5, 13, 14, 21). 

*T. ptyseos* has been isolated from other materials such as soil, poultry carcasses, edible microalgae, water supplies, beef and pineapple in different developing and developed countries (22-25). *T. ptyseos* infections should alert microbiologists and medical physicians about the potential pathogenicity of this agent in different clinical wards (1, 5). The virulence factors and pathogenesis of this organism is unknown. Neonates (first 28 days), particularly pre-term, low birth weight or immunocompromised infants, people with diabetes, and neuromuscular disease patients, including ataxia telangiectasia patients, using feeding tubes are at highest risk for *T. ptyseos* infection.

The results of the current study showed that all *T. ptyseos* strains isolated from PIF samples were sensitive to most of the tested antibiotics. In the present study, isolates were all resistant to ampicillin, carbenicillin, cotrimoxazole and amoxicillin equally. *T. ptyseos* resistance to ampicillin and cotrimoxazole has been previously reported by different investigators. Probably, antibiotic resistant profile of *T. ptyseos* differs geographically. *T. ptyseos* ability to survive and grow in PIF products raises public health concern especially between susceptible persons. Contamination of formula with *T. ptyseos* can occur in various steps including: during preparation, handling, and storage of reconstituted products. This food-borne pathogen can enter the infant milk formula with raw material used for producing the PIF. The contamination of the powdered infant formula milk or other dry ingredients can even occur after pasteurization process.

Great care should be exercised to maintain proper hygienic conditions during preparation process and handling of raw milk and milk products. Raw milk should be properly pasteurized or boiled before consumption. Manufacturers warning labels on powdered infant formula packages should emphasize that these products are non-sterile and require proper preparation, handling, and storage (26). Only a few developed countries have reported the infections caused by *T. ptyseos* due to use of contaminated powdered infant formula milk. There is a probability of significant underreporting of infections in all countries. The absence of reports is likely due to the limitations of the current surveillance systems in most countries and the lack of notification of the problem rather than an absence of the disease. 

Since infant formula is widely used, the presence of *T. ptyseos* in it can be a serious public health difficulty in most developed and developing countries because of its life threatening infections in infants, the elderly, and immunocompromised persons; infants of human immunodeficiency virus (HIV)- positive mothers are also at risk both, because they may specifically require infant formula and may be more susceptible to infection. The results of accurate surveillance should be used to reconsider guided policies to prevent contamination of powdered infant formula with *T. ptyseos*, and subsequently the severe infections related to this opportunistic pathogen. 

The present study was the first report on the isolation and identification of *T. ptyseos* in powdered PIF in Iran. There is no information on previous isolation of this organism from food samples and/or clinical specimens in this country. The findings of the current study revealed the presence of *T. ptyseos* in the PIF consumed in Iran. *T. ptyseos* is frequently present in various foods; therefore, investigation on these samples is required. Tracking *T. ptyseos* in a wide variety of foods and also immunocompromised people such as HIV-positive patients is important to reveal the possible transmission routes of this pathogen to the others. Further molecular studies are required to determine the genetic relationship between *T. ptyseos* strains isolated from different sources.

*T. ptyseos* is relatively unknown to many clinical and food microbiology laboratory workers and can be easily missed in the routine diagnosis of clinical human specimens and food samples (e.g. powdered infant milk formula). However since the classification, nomenclature and identification of *T. ptyseos* has been already established, it is hoped that laboratory workers will become aware and lookout for this bacterium in the food samples and clinical specimens (especially specimens collected from newborns and immunosuppressed patients). It is also hoped that more investigations on the epidemiology and the pathogenesis of *T. ptyseos* will be available to obtain a better understanding and comprehension of its clinical significance.

## References

[1] Hollis DG, Hickman FW, Fanning GR, Farmer JJ, Weaver RE, Brenner DJ (1981). Tatumella ptyseos gen. nov., sp. nov., a member of the family Enterobacteriaceae found in clinical specimens.. J Clin Microbiol..

[2] Kageyama B, Nakae M, Yagi S, Sonoyama T (1992). Pantoea punctata sp. nov., Pantoea citrea sp. nov., and Pantoea terrea sp. nov. isolated from fruit and soil samples.. Int J Syst Bacteriol..

[3] Farmer JJ, Davis BR, Hickman-Brenner FW, McWhorter A, Huntley-Carter GP, Asbury MA (1985). Biochemical identification of new species and biogroups of Enterobacteriaceae isolated from clinical specimens.. J Clin Microbiol..

[4] Marín‐Cevada V, Caballero‐Mellado J, Bustillos‐Cristales R, Muñoz‐Rojas J, Mascarúa‐Esparza MA, Castañeda‐Lucio M (2010). Tatumella ptyseos, an unrevealed causative agent of pink disease in pineapple.. J Phytopathol..

[5] Costa PS, Mendes JM, Ribeiro GM (2008). Tatumella ptyseos causing severe human infection: report of the first two Brazilian cases.. Braz J Infect Dis..

[6] Brady CL, Venter SN, Cleenwerck I, Vandemeulebroecke K, De Vos P, Coutinho TA (2010). Transfer of Pantoea citrea, Pantoea punctata and Pantoea terrea to the genus Tatumella emend. as Tatumella citrea comb. nov., Tatumella punctata comb. nov. and Tatumella terrea comb. nov. and description of Tatumella morbirosei sp. nov.. Int J Syst Evol Microbiol..

[7] Hamana K (1996). Distribution of diaminopropane and acetylspermidine in Enterobacteriaceae.. Can J Microbiol..

[8] Ramia S, Neter E, Brenner DJ (1982). Production of enterobacterial common antigen as an aid to classification of newly identified species of the families Enterobacteriaceae and Vibrionaceae.. Int J Systematic Bacteriol..

[9] Sproer C, Mendrock U, Swiderski J, Lang E, Stackebrandt E (1999). The phylogenetic position of Serratia, Buttiauxella and some other genera of the family Enterobacteriaceae.. Int J Syst Bacteriol..

[10] Brady C, Cleenwerck I, Venter S, Vancanneyt M, Swings J, Coutinho T (2008). Phylogeny and identification of Pantoea species associated with plants, humans and the natural environment based on multilocus sequence analysis (MLSA).. Syst Appl Microbiol..

[11] Tan SC, Wong YH, Jegathesan M, Chang SM (1989). The first isolate of Tatumella ptyseos in Malaysia.. Malays J Pathol..

[12] Agbaje M, Dipeolu MA, Oyekunle MA, Grace D, Ojo OE (2013). Isolation of Tatumella ptyseos from Beef in Ibadan, Nigeria.. Niger Vet J..

[13] Stone ND, O'Hara CM, Williams PP, McGowan JJ, Tenover FC (2007). Comparison of disk diffusion, VITEK 2, and broth microdilution antimicrobial susceptibility test results for unusual species of Enterobacteriaceae.. J Clin Microbiol..

[14] Berktaş M., Uzun K., Bozkurt H., Kurtoglu M.G., Guducuoglu H., Aydin S. (2013). Pulmonary infection of Tatumella ptyseos developed on the background of pulmonary tuberculosis.. East J Med..

[15] Nisiotou AA, Rantsiou K, Iliopoulos V, Cocolin L, Nychas GJ (2011). Bacterial species associated with sound and Botrytis-infected grapes from a Greek vineyard.. Int J Food Microbiol..

[16] Rodríguez MC, Navarrete JM (2001). Bacteriological study and determination of sensitivity to 21 antibiotics, and patient population treated at the General Hospital of Mexico in 1999.. Enf Infec Y Micro..

[17] Carlisle EM, Poroyko V, Caplan MS, Alverdy JA, Liu D (2011). Gram negative bacteria are associated with the early stages of necrotizing enterocolitis.. PLoS One..

[18] (2003). Food and Drug Administration, 2002a. Isolation and enumeration of Enterobacter sakazakii from dehydrated powdered infant formula..

[19] (2003). U.S. Food and Drug Administration, 2002b. Questions and answers on method for E. sakazakii in powdered infant formula..

[20] (2011). Clinical and Laboratory Standards Institute (CLSI)..

[21] Janda JM, Abbott SL, Janda MJ (2006). The enterobacteria..

[22] Silva V, Dias E, Vale R, Silva R, Moreira GF (2007). Isolamento e identificação de bactérias presentes nos solos de cobertura utilizados no cultivo do cogumelo Agaricus blazei Murril.. Agrotec..

[23] Hiroshi N, Atsuhito E, Norihito T (2006). 26:45-49 . Browning discoloration by reheating of foods in container-I: Isolation and identification of causal bacteria of browning discoloration of natudaidai.. Toyo Jr College Food Tech Toyo Inst Food Tech..

[24] Jimenez SM, Tiburzi MC, Salsi MS, Pirovani ME, Moguilevsky MA (2003). The role of visible faecal material as a vehicle for generic Escherichia coli, coliform, and other enterobacteria contaminating poultry carcasses during slaughtering.. J Appl Microbiol..

[25] Moore JE, Xu J, Millar BC (2002). Diversity of the microflora of edible macroalga (Palmaria palmata).. Food Microbiol..

[26] Mardaneh J, Dallal MM (2013). Isolation, identification and antimicrobial susceptibility of Pantoea (Enterobacter) agglomerans isolated from consumed powdered infant formula milk (PIF) in NICU ward: First report from Iran.. Iran J Microbiol..

